# The function of apolipoproteins L (APOLs): relevance for kidney disease, neurotransmission disorders, cancer and viral infection

**DOI:** 10.1111/febs.15444

**Published:** 2020-06-25

**Authors:** Etienne Pays

**Affiliations:** ^1^ Laboratory of Molecular Parasitology IBMM Université Libre de Bruxelles Gosselies Belgium

**Keywords:** actomyosin organization, apoptosis, autophagy, mitochondrial fission, vesicular trafficking

## Abstract

The discovery that apolipoprotein L1 (APOL1) is the trypanolytic factor of human serum raised interest about the function of APOLs, especially following the unexpected finding that in addition to their protective action against sleeping sickness, APOL1 C‐terminal variants also cause kidney disease. Based on the analysis of the structure and trypanolytic activity of APOL1, it was proposed that APOLs could function as ion channels of intracellular membranes and be involved in mechanisms triggering programmed cell death. In this review, the recent finding that APOL1 and APOL3 inversely control the synthesis of phosphatidylinositol‐4‐phosphate (PI(4)P) by the Golgi PI(4)‐kinase IIIB (PI4KB) is commented. APOL3 promotes Ca^2+^‐dependent activation of PI4KB, but due to their increased interaction with APOL3, APOL1 C‐terminal variants can inactivate APOL3, leading to reduction of Golgi PI(4)P synthesis. The impact of APOLs on several pathological processes that depend on Golgi PI(4)P levels is discussed. I propose that through their effect on PI4KB activity, APOLs control not only actomyosin activities related to vesicular trafficking, but also the generation and elongation of autophagosomes induced by inflammation.

## Graphical abstract

APOL1 and APOL3 are K^+^ channels of *trans*‐Golgi and endoplasmic reticulum membranes. Ca^2+^‐dependent binding of APOL3 to neuronal calcium sensor 1 (NCS‐1) promotes the interaction of NCS‐1 with PI(4)‐kinase IIIB (PI4KB) and consecutive PI4KB activation, and APOL1 can antagonize this process. Since the synthesis of phosphatidylinositol‐4‐phosphate (PI(4)P) by PI4KB influences vesicular trafficking, mitochondrial fission and autophagy, I discuss the involvement of APOLs in several pathological processes that depend on PI(4)P levels, particularly kidney disease and viral infection.

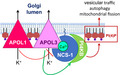

**Abbreviations**
APOL: apolipoprotein L
BH3: BCL2 homology domain 3 sequence
ER: endoplasmic reticulum
HC: hydrophobic cluster
ID: interacting domain
LZ: leucine zipper
MAD: membrane‐addressing domain
MERCs: ER/mitochondrion contact sites
PFD: pore‐forming domain
PI(4)P: phosphatidylinositol‐4‐phosphate
PI4KB: PI(4)‐kinase IIIB
SRID: SRA‐interacting domain

## Introduction

Since the identification of APOL1 as component of serum high‐density lipoprotein particles [1], the nature of the functions performed by the members of the human apolipoprotein L (APOL) family remained elusive. Salient features included the evidence that the only secreted member of this family, the primate‐specific APOL1, protects humans against the African parasite *Trypanosoma brucei*, whereas natural APOL1 C‐terminal variants termed G1 and G2, which also protect against the sleeping sickness agent *Trypanosoma rhodesiense*, can cause kidney disease [2, 3, 4]. In addition, a role linked to viral infection was suggested for APOL1 and APOL3, given the strong induction of their expression through the viral inflammatory TLR3/TRIF pathway [5, 6], and the linkage of G1‐ or G2‐induced kidney disease with HIV infection [7]. Accordingly, in both dendritic cells and kidney podocytes, APOL1 and APOL3 were found to be involved in apoptosis triggered by the viral mimetic agent poly(I:C) [6, 8], and in macrophages, APOL1 was reported to restrict HIV‐1 infection [9]. In trypanosomes, the mechanism of lysis by APOL1 resembled apoptosis initiated by the APOL1 membrane pore‐forming activity [10], and recombinant APOL3 exhibited both lytic and pore‐forming activities like APOL1 [8, 11]. Therefore, the death‐promoting activity of APOL1 and APOL3 could be related to their potential for ion transport across intracellular membranes. Such conclusion was supported by the evidence that apoptotic BCL2 family members also possess an ion pore‐forming activity, and a seemingly functional apoptotic BCL2 homology domain 3 sequence (BH3) is present in APOLs [6, 10, 12].

Further study of APOL function concentrated on the role of APOL1 in kidney podocyte biology, trying to understand how the G1 and G2 variants trigger podocyte dysfunction. Despite a large number of publications, reviewed in Ref. [13, 14], no precise mechanism could be identified except for evidence of pleiotropic effects involving altered vesicular trafficking. In these studies, a major drawback was the nonspecific toxicity induced by ectopic expression of APOL1 or APOL1 variants [15], presumably linked to uncontrolled pore‐forming activity. Another issue was the uncertainty about the intracellular localization of APOL1. APOL1 was identified as a secreted lipoprotein [1], and indeed, the APOL1 gene is the only one of the APOL family to encode a protein with a signal peptide (SP) [12]. Thus, APOL1 must be present in the luminal compartment of the cellular secretory pathway. However, alternative mRNA splicing generates transcripts encoding APOL1 isoforms that do not appear to contain an efficient SP, suggesting the possible presence of APOL1 also in the podocyte cytosol [8, 16]. When taking both the toxicity and intracellular localization of APOL into consideration, a primary function for APOL1 and APOL3 was recently identified: the control of phosphatidylinositol‐4‐phosphate (PI(4)P) synthesis at the *trans*‐Golgi network [8]. The aim of this review was to discuss the implications of this finding in terms of diseases influenced by PI(4)P levels.

## APOL1 and APOL3 activities

### Ion pore‐forming activity, trypanolysis and toxicity

**Fig. 1:**
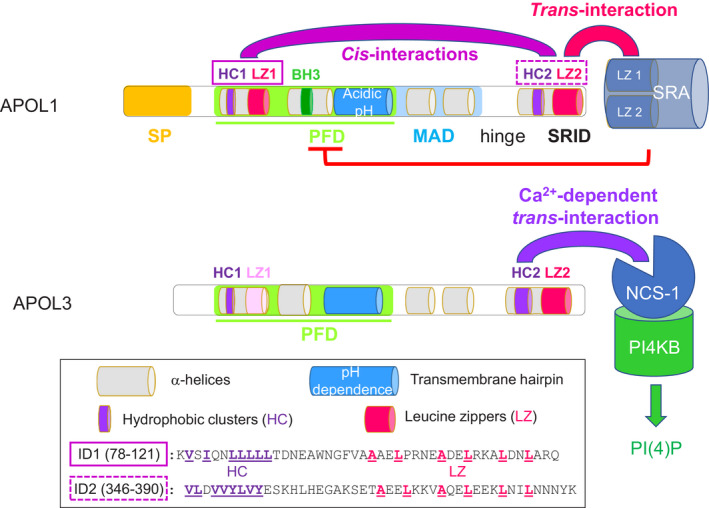
Structure and function of APOL1 and APOL3 (398 and 331 amino acids, respectively). Whereas transmembrane insertion of the APOL1 hairpin depends on acidic pH, that of APOL3 does not [11]. In APOL1, *cis*‐interactions occur between two interacting domains (IDs) that each contains a tandem of HC and LZ [8]. The *T. rhodesiense* protein SRA interacts with APOL1 in the parasite endocytic compartment, through coiled‐coiling of its N‐terminal LZ with the C‐terminal LZ (LZ2) of APOL1. This interaction neutralizes APOL1 trypanolytic activity, which is linked to transmembrane ion transport by the PFD [2, 10, 17]. SRA binding to LZ2 also inhibits APOL1 toxicity in *E. coli* [25]. APOL3 exhibits Ca^2+^‐dependent binding of high affinity to NCS‐1, which promotes interaction of NCS‐1 with PI4KB, stimulating PI(4)P synthesis at the Golgi [8].

**Fig. 2:**
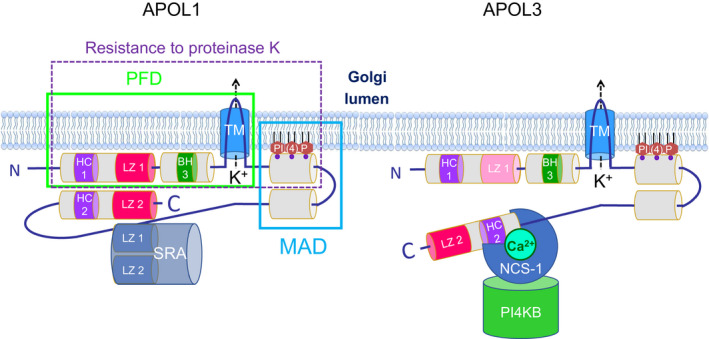
Model structures of APOL1 and APOL3, and their interactions with other proteins. The PFD and MAD of APOL1 were defined in Ref. [17], whereas the delimitation of the APOL1 domain protected against *in vitro* digestion by proteinase K results from fig. 9 data in Ref. [10]. MAD interaction with PI(4)P is hypothetical. See Fig. 1 for abbreviations. From structural predictions [17, 27], the two helices containing the HC/LZ pairs could each be folded in a double‐stranded hairpin.

Based on results obtained following APOL1 expression in different cell types such as *Escherichia coli*, *Saccharomyces cerevisiae* and *T. brucei,* as well as *in vitro* analyses, a tentative structure was proposed for APOL1 [17]. This structure is shared by APOL3, albeit with significant differences [11]. As schematized in Figs 1 and 2, an N‐terminal SP sequence is present in APOL1 only, but APOL1 and APOL3 contain a similar pore‐forming domain (PFD), and both proteins exhibit a similar trypanolytic potential, although differing in their pH dependence [11]. In planar lipid bilayers at neutral pH, APOL1 and APOL3 respectively generated voltage‐dependent and voltage‐independent K^+^‐selective channels [8, 18]. In both cases, the PFD includes a transmembrane hairpin (TM) able of *in vitro* insertion into mitochondrial membranes [10], which bears similarity to the hairpin present in the PFD of bacterial colicins, diphtheria toxin and BCL2 family members [12]. Accordingly, mutagenesis of the short loop of this hairpin in either APOL1 or APOL3 inactivated both their trypanolytic activity and toxicity in *E. coli* or yeast [8, 11]. The functional importance of this TM was also revealed when comparing the trypanolytic potential and toxicity between APOL1 and APOL3. The pH dependence of this activity strikingly differed between APOLs, since APOL1 required acidic pH for trypanolysis and toxicity in *E. coli*, whereas APOL3 did not [11]. This could be ascribed to the presence of acidic amino acids in the TM of APOL1 only, which impede membrane insertion at neutral pH. Accordingly, introducing these residues into the APOL3 hairpin conferred acidic pH dependence of APOL3 trypanolysis and toxicity [11]. In addition to their differential pH dependence for membrane insertion, APOL1 and APOL3 also exhibited differences between their ion‐conducting functions, since the ion selectivity was affected by pH conditions only for APOL1 (see next paragraph). Therefore, as also true for colicins, the TM of APOL1 and APOL3 is central to the ability of these proteins to transport ions and induce cellular toxicity. However, again like in colicins, the APOL1 PFD also includes several alpha‐helices in addition to the TM. In particular, a helix immediately preceding the TM contains a sequence bearing similarity to the BH3 motif of apoptotic BCL2 [12] (Figs 1 and 2). This sequence could be truly functional as BH3‐interacting domain, since site‐directed mutagenesis known to disrupt selectively the BH3 activity of murine BAX affected the trypanolytic potential of APOL1 [10], and in mouse dendritic cells, the mouse APOLs7 involvement in poly(I:C)‐induced apoptosis seemed to involve interaction of the BH3‐like peptide with the antiapoptotic protein BCL‐XL [6]. Of note, BCL‐XL‐interacting proteins with a BH3‐like motif exist in trypanosomatids [19]. So far, the possible involvement of such proteins in trypanolysis by APOL1 has not been tested.

### The dual nature of the APOL1 pore

Once inserted into synthetic membranes, the pore‐forming activity of APOL1 is strongly enhanced by switching from acidic to neutral conditions, which also switches the ion transport preference from anions to cations [18]. These characteristics could be related to the different steps of the intracellular travel of APOL1 within the trypanosome. APOL1 is internalized with HDLs via receptor‐mediated uptake in the flagellar pocket of the parasite [20], and delivered to acidic endosomal compartments where the low pH allows membrane insertion of the protein [2]. This triggers permeabilization of endolysosomal membranes and transmembrane flux of chloride ions that leads to lysosome swelling [10, 17]. However, this swelling in itself is not responsible for trypanolysis [10]. Trypanolysis rather results from kinesin‐mediated trafficking of some APOL1‐containing endosomal membranes to the mitochondrion [10]. This is linked to mitochondrial membrane permeabilization that allows a parasite endonuclease (TbEndoG) to exit the mitochondrial membrane compartment and reach the nucleus, degrading the DNA in nucleosomal units like occurs during apoptosis in higher eukaryotes [10]. It is tempting to speculate that efficient cation transport induced by the APOL1 pores at neutral pH in the mitochondrial membrane is involved in this apoptosis‐like process, but BH3 interactions could alternatively be responsible. Incidentally, APOL1 also inhibits the fission of the mitochondrial membrane, in a process mimicked by down‐regulation of the trypanosome homologue of the dynamin‐1‐like protein DRP1 (TbMFNL), but this effect is independent of trypanolysis [10].

While in trypanosomes, the APOL1 dependence on acidic pH for membrane insertion can be explained by the function of this protein in the endolysosomal compartment, APOL1 could not be detected in this compartment of APOL1‐producing cells such as kidney podocytes [8]. Like APOL3, APOL1 rather exhibits intranuclear and perinuclear distributions, the latter involving important colocalization with endoplasmic reticulum (ER) and *trans*‐Golgi markers [8]. Moreover, APOL1 largely shares the membrane association characteristics of intracellular integral transmembrane proteins such as the ER protein calnexin [8], implying that unknown mechanisms allow intracellular membrane insertion of APOL1 at neutral pH. The presence of APOL1 in the nucleus and cytosol, thus outside of the secretory pathway, is linked to distinct protein processing of intracellular APOL1 as compared to secreted APOL1 [8]. Among different APOL1 isoforms resulting from alternative transcript splicing, APOL1 isoform 3, which is identical to APOL1 except that it is not predicted to contain a functional SP, could account for the intracellular component of APOL1 [8, 16].

In summary, two distinct APOL1 isoforms could account for APOL1 biology within and outside of the secretory compartment, and the acidic pH dependence of APOL1 insertion in membranes would be restricted to the trypanosome intracellular environment.

### Control of the APOL1 pore‐forming activity

While the pore‐forming activity of APOL1 undoubtedly depends on membrane insertion of the TM, this activity appears to be controlled by protein domains located downstream from the PFD.

In functional tests, both in *E. coli* and in trypanosomes, a region that was termed ‘membrane‐addressing domain’ (MAD; see Figs 1 and 2) was found to be required for toxicity [17]. According to a speculative model, this region could be folded in a pH‐sensitive helical hairpin, contributing to anchor APOL1 to membranes owing to the pH dependence of its hydrophobic character [17]. Whether this region could modulate *in vivo* APOL1 access to membranes is not known, but, together with the contiguous PFD, it was partially protected against digestion by proteinase K upon *in vitro* interaction of APOL1 with isolated mitochondrial membranes [10] (Fig. 2), indicating that the MAD is tightly associated with the membrane. As a possible explanation, the MAD could be involved in the demonstrated APOL1 ability to bind anionic lipids such as phosphoinositides, phosphatidic acid and cardiolipin [8] (Fig. 2). Since cardiolipin promotes the transmembrane insertion of the BCL2 family member BAX [21, 22], such binding could account for APOL1 PFD insertion into the cardiolipin‐rich membrane of *E. coli* or mitochondria, even at neutral pH [10, 17]. Similarly, binding to phosphatidic acid could possibly favour APOL1 PFD insertion in Golgi membranes, because phosphatidic acid directs both Golgi fission and transmembrane insertion of the BCL2 family member tBID [23, 24]. As observed for BAX and tBID [21, 22, 24], these phospholipids could also promote the APOL oligomerization necessary for PFD activity.

Further downstream, a C‐terminal leucine zipper (LZ) helix was found to be required for both trypanolytic and *in vitro* pore‐forming activities, because deletion of this LZ helix inactivates the APOL1 PFD [18, 25]. Interestingly, this helix is selectively targeted by the *T. rhodesiense* serum resistance‐associated (SRA) protein. SRA neutralizes APOL1 toxicity following direct coiled‐coiling interaction of its N‐terminal LZ helix with the C‐terminal LZ helix of APOL1, and thus allows the *T. rhodesiense* parasite to grow in humans, causing sleeping sickness in eastern Africa [2, 26, 27]. Binding of SRA to the C‐terminal helix region, termed SRID in Fig. 1 (SRA‐interacting domain), can prevent APOL1 toxicity even in *E. coli* [25]. In return, mutations in the SRID LZ helix can confer on APOL1 the ability to kill *T. rhodesiense* because these mutant APOL1 variants resist the neutralizing interaction with SRA [3, 25]. Thus, either the absence of the SRID LZ helix or the binding of SRA to this region equally inhibits the pore‐forming activity of APOL1 (Fig. 1).

Apart from its *trans*‐interaction with SRA, the SRID LZ helix also interacts in *cis* with another LZ helix situated in the N‐terminal region [8] (Figs 1 and 2). The N‐ and C‐terminal LZs were termed LZ1 and LZ2, respectively. Like occurs for the trypanosome variant surface glycoprotein family, to which SRA belongs, the multicoil interaction ability of LZs can allow more than a single LZ‐to‐LZ binding [27]. Thus, APOL1 LZ2 could interact with both SRA N‐terminal LZ helix and APOL1 LZ1. In APOL1, each LZ is preceded by a cluster of hydrophobic residues [HC1 and HC2, for the N‐ and C‐terminal hydrophobic clusters (HC), respectively]. Interaction between these HCs also contributes to strengthen the *cis*‐interactions within APOL1 [8]. In APOL3, no evidence for *cis*‐interaction was obtained, presumably due to the poor coiling capacity of LZ1 in this case [8]. As APOL3 can exhibit pore‐forming activity either in synthetic membranes or in trypanosomes [8, 11], *cis*‐interactions between the HC1/LZ1 and HC2/LZ2 pairs are not required for this activity. As discussed below, *cis*‐interactions in APOL1 may result from the necessity to prevent interactions between APOL1 and APOL3 in order to avoid cellular dysfunctions.

### Control of PI4KB activity

Using the yeast two‐hybrid system to identify APOL‐interacting proteins, APOL3 was found to exhibit high affinity and Ca^2+^‐dependent binding to neuronal calcium sensor 1 (NCS‐1) [8]. NCS‐1 is a regulator of calcium signalling, structured in two lobes each containing two Ca^2+^‐binding EF‐hands, the most N‐terminal one being nonfunctional [28]. Ca^2+^ binding triggers NCS‐1 conformational modification, exposing a hydrophobic crevice for interactions with different proteins, as particularly detailed in the case of activation of the yeast PI(4)‐kinase IIIB (PI4KB) homologue phosphatidylinositol 4‐kinase 1 (PIK1) by the yeast NCS‐1 homologue frequenin 1 (FRQ1) [29]. In keeping with evidence that interactions of different proteins with NCS‐1 rely on hydrophobic contacts [30], APOL3 binding to NCS‐1 involves HC2 [8] (Fig. 1). That APOL3 interacts with NCS‐1 is also consistent with the propensity of NCS‐1 to interact with cation channels [31, 32, 33]. Interestingly, APOL3 binding to NCS‐1 strongly promoted NCS‐1 interaction with PI4KB and stimulated PI4KB activity [8] (Fig. 1). As expected from these observations, APOL3 KO resulted in reduction of PI(4)P levels of podocytes [8]. Conversely, APOL1 did not bind to NCS‐1, but appeared to negatively influence PI(4)P levels, probably through indirect PI4KB inhibition [8].

In yeast, PIK1 activity clearly depends on interaction with FRQ1, and can still occur upon replacement of FRQ1 with NCS‐1 [34]. In these cells, activation of PI(4)P synthesis by the PI4KB homologue seems to be the only function ascribed to the NCS‐1 homologue [29, 34], suggesting that the primary role of NCS‐1 is the control of PI4KB. In human cells, both interaction of NCS‐1 with PI4KB and resulting PI4KB activation are supported by strong *in vivo* evidence, but without clear demonstration *in vitro* [8, 35, 36, 37, 38, 39, 40]. In particular, despite evidence for Ca^2+^ dependence of *in vivo* PI4KB activation by NCS‐1 [36, 37, 38, 39, 40], like also occurs for PIK1 activation by FRQ1 [29, 34, 41], no such clear dependence was observed *in vitro* [8, 35]. The involvement of N‐myristoylation was invoked in this process, but only little evidence supported this hypothesis [35, 38, 41]. In this context, the finding that Ca^2+^‐dependent interaction of APOL3 with NCS‐1 promotes NCS‐1 binding to PI4KB irrespective of NCS‐1 N‐myristoylation [8] provides a possible explanation. Thus, APOL3 could trigger Ca^2+^‐dependent PI4KB activation through promotion of NCS‐1 binding to PI4KB (Figs 1 and 2). Under *in vitro* conditions, APOL3 stimulates recombinant PI4KB activity, whereas NCS‐1 does not [8]. Since there is no evidence for interaction between APOL3 and PI4KB unless NCS‐1 is added [8], it is possible that despite its ability to directly activate PI4KB *in vitro*, *in vivo* APOL3 should interact with NCS‐1 to activate PI4KB.

Further evidence that APOLs control PI4KB activity was provided by a recent finding (Hennig D, Graversen JH & Pays E, unpublished). We found that APOL3 also binds with high affinity to calneuron 1 (CALN‐1), a PI4KB inhibitor [40], but only at low Ca^2+^ concentrations, thus under opposite Ca^2+^ conditions as compared to NCS‐1. Therefore, depending on cytosolic Ca^2+^ levels at the periphery of ER and *trans*‐Golgi membranes, APOL3 could interact with either NCS‐1 or CALN‐1, respectively, activating or inhibiting PI(4)P synthesis by PI4KB.

In APOL1+3 double KO cells, paradoxically, PI4KB appeared to be active despite APOL3 absence. As discussed in the next paragraph, this could possibly be explained by the effect of APOL1 on cytosolic Ca^2+^ levels.

### Control of Ca^2+^ uptake in ER and Golgi stores

In addition to their effects on Golgi PI(4)P synthesis by PI4KB, and possibly linked to the control of these effects, APOL1 and APOL3 also influence the Ca^2+^ level in ER and Golgi stores [8]. Both proteins contribute to increase Ca^2+^ uptake. Since the activity of some other low conductance K^+^ channels also promotes Ca^2+^ uptake [42], Ca^2+^ storage by APOLs may be linked to the capacity of these proteins to transport K^+^ from the cytosol to the lumen of intracellular Ca^2+^ stores. Interestingly, APOL3 interacts with two Ca^2+^‐binding proteins that not only conversely control PI4KB, but also conversely control Ca^2+^ storage. Whereas NCS‐1 controls Ca^2+^ efflux through activation of the inositol 1,4,5‐trisphosphate receptor (IP3R) [32], CALN‐1 controls Ca^2+^ influx possibly through activation of the sarcoendoplasmic reticulum calcium transport ATPase [43]. Thus, the activity of APOLs may be involved in the Ca^2+^ signalling pathways that regulate cellular physiology depending on environmental conditions.

Curiously, in podocytes the absence of APOL3 alone led to PI4KB inactivation, whereas the absence of both APOL1 and APOL3 restored PI4KB activity [8]. Since the only difference between APOL3KO and APOL1+3KO cells is the absence of APOL1 in the latter case, it appears that in the absence of APOL3, APOL1 inhibits PI4KB activation by NCS‐1. Perhaps in line with this hypothesis, APOL3KO cells, but not APOL1+3KO cells, exhibited increased Ca^2+^ uptake in intracellular stores [8]. Thus, in the first case only, the absence of APOL3 may promote increased cytosolic Ca^2+^ depletion by APOL1, thus favouring CALN‐1 over NCS‐1 activity [40], which is expected to result in PI4KB inhibition. The removal of APOL1 in APOL1+3KO cells would relieve this effect, restoring Ca^2+^‐dependent stimulation of PI4KB by NCS‐1. However, how in this case NCS‐1 would activate PI4KB without APOL3 remains a mystery. As an alternative possibility, the only effect of APOL3 on PI4KB activity would be to condition its Ca^2+^‐dependent activation through NCS‐1 binding. In APOL3 absence, PI4KB activity would be both NCS‐1‐ and Ca^2+^‐independent, but more susceptible to inhibition by APOL1. Clearly, the mechanism for *in vivo* activation of PI4KB at the Golgi involves components and processes that still need to be identified.

### Key role of PI(4)P in cellular physiology

**Fig. 3:**
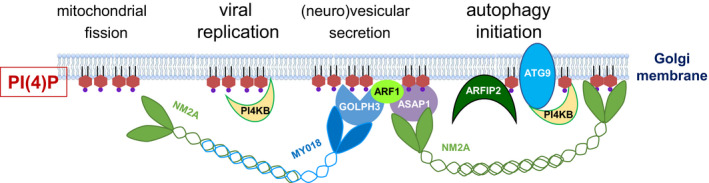
Role of PI(4)P in various processes depending on intracellular membrane remodelling. Induction of membrane curvature and membrane recruitment of actomyosin characterize the different processes listed above the scheme.

NCS‐1‐mediated activation of PI(4)P synthesis by PI4KB clearly stimulates vesicular secretion from the *trans*‐Golgi, membrane trafficking to the plasma membrane and exocytosis [35, 36, 37, 38, 39, 40]. Indeed, PI(4)P exerts a key role on several processes that depend on membrane curvature, such as the formation of secretory vesicles, autophagic vesicles and virus‐induced membranous replicative platforms, as well as the fission of organelle membranes. In addition to the intrinsic potential of PI(4)P to induce membrane curvature [44, 45], Golgi PI(4)P‐binding proteins such as Golgi phosphoprotein 3 (GOLPH3) and the phosphoinositide‐binding GTPase‐activating protein Arf‐GAP with SH3, ANK and PH domains (ASAP1) contribute to trigger membrane bending necessary for fission, secretion and trafficking [45, 46] (Fig. 3).

Multiple pathways connect PI(4)P with the secretion and fission motor nonmuscle myosin 2A (NM2A) (Fig. 3). For example, (a) the PI(4)P‐binding protein GOLPH3 binds to the NM2A‐interacting myosin MYO18A [47, 48, 49, 50], (b) ASAP1 interacts with the GOLPH3‐binder ADP‐ribosylation factor 1 and with NM2A [51, 52, 53, 54], (c) the NM2A heavy chain MYH9 directly binds to anionic phospholipids [55], and (d) together with autophagy‐related protein 9 (ATG9) and arfaptin 2, PI4KB and PI(4)P initiate autophagy in NM2A‐driven vesicles derived from the *trans*‐Golgi [56]. Given the requirement of PIK1 and ATG9 for autophagy initiation also in *S. cerevisiae* [57], Golgi PI(4)P‐mediated autophagy is probably an evolutionary ancient process.

Thus, affecting Golgi PI(4)P level strongly influences actomyosin organization for secretion, autophagy and vesicular trafficking (review in Ref. [58]). Moreover, it was recently found that Golgi PI(4)P synthesis by PI4KB is required for mitochondrial fission at ER contact sites, where actomyosin and the DRP1 dynamin constrict the organelles [59]. Thus, Golgi PI(4)P is not only involved in vesicular trafficking, but also involved in mitochondrial fission.

Given their impact on PI(4)P levels, APOL1 and APOL3 are likely to be involved in the organization of actomyosin activity for membrane remodelling, such as annular membrane contraction for vesicle budding or fission. Here, I will briefly discuss the involvement of APOLs in diseases related to such processes.

## APOLs and kidney disease

### Disease induced by the APOL1 variants G1 and G2

Alteration of the APOL1 C‐terminal LZ helix, such as occurs in natural G1 and G2 variants, triggers kidney disease [3, 4]. Numerous studies were dedicated to deciphering the molecular mechanism at play, but apart from evidence for disturbances in vesicular trafficking and/or autophagy, until recently no precise explanation could be proposed (reviews in Ref. [13, 14]). Established facts include the following: (a) APOL1 variants present intracellularly must be involved in podocyte dysfunction, because circulating APOL1 levels do not correlate with the disease, and because poor kidney allograft outcomes are associated with the APOL1 genotype of the transplanted kidney, but not of the recipient patient [13]; (b) individuals lacking APOL1 due to mutations in both *APOL1* alleles [60] appear to be healthy [61]. Therefore, kidney disease must result from negative intracellular effects of the APOL1 variants, rather than APOL1 inactivation. In accordance with this conclusion, mice, which do not have the APOL1 gene, exhibit kidney disease following transgenic expression of human APOL1 C‐terminal variants, and not wild‐type APOL1 [4]. Therefore, it can be safely concluded that distortion of the C‐terminal LZ of APOL1 must induce negative effects distinct from APOL1 inactivation.

### Recent experimental model [8]

In cultured human podocytes, either truncation of the APOL1 C‐terminal LZ (in APOL1Δ cells) or deletion of APOL3 (in APOL3KO cells) induced a similar reorganization of actomyosin activity, with reduced content of stress fibres, reduced cellular size and adherence, increased motility and increased organelle fission [8]. In keeping with these observations, APOL1 was found to be associated with NM2A and other actomyosin components such as tropomyosin and gelsolin [8]. The actomyosin phenotype of both APOL1Δ and APOL3KO cells was linked to strong reduction of Golgi PI(4)P levels, indicative of PI4KB inactivation [8]. In line with this finding, APOL1 and APOL3 were found to significantly colocalize with PI(4)P, PI4KB, the PI4KB activator NCS‐1 and the PI(4)P‐binding protein GOLPH3 [8]. Moreover, in the presence of Ca^2+^, APOL3 exhibited high‐affinity binding to NCS‐1, and this binding strongly promoted interaction of NCS‐1 with PI4KB [8], which is linked to *in vivo* PI4KB activation [36, 37, 38, 39, 40]. APOL3 was also able to stimulate *in vitro* PI4KB activity [8]. Either mutagenesis or deletion of the APOL1 C‐terminal helix induced strong increase in APOL1 interaction with APOL3, and this interaction both hindered the interaction between APOL3 and NCS‐1 and inhibited the ion channel activity of the APOL3/NCS‐1 complex [8].

**Fig. 4:**
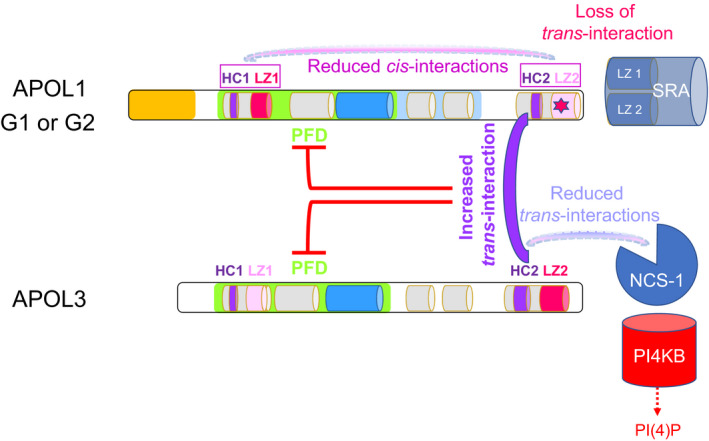
Induction of APOL1 interaction with APOL3 in the G1 and G2 variants. The red asterisk in APOL1 LZ2 symbolizes G1 or G2 mutations. The G1 and G2 mutations in the C‐terminal LZ2 of APOL1 inhibit the neutralizing interaction with SRA, providing to these APOL1 variants the ability to kill *T. rhodesiense* [3]. However, they also reduce *cis*‐interactions, exposing the APOL1 HCs. Interactions between APOL1 and APOL3 HCs trigger inactivation of both proteins and interfere with APOL3/NCS‐1 interactions, thereby preventing stimulation of PI4KB activity [8].

Taken collectively, these observations indicated that APOL1 C‐terminal variants inhibit APOL3 activity through increased interaction with this protein, resulting in inhibition of PI(4)P synthesis by PI4KB (Fig. 4). This conclusion provides a straightforward explanation for the mimicking of this phenotype following APOL3 KO, and also accounts for the lack of altered phenotype in APOL1KO podocytes.

How C‐terminal alteration of APOL1 triggers increased interaction with APOL3 and consecutive APOL3 inactivation could result from interference with *cis*‐interactions that normally take place between the N‐ and C‐terminal APOL1 LZ helices [8]. Indeed, reducing these interactions following either LZ2 loss or LZ2 disruption resulted in increased exposure of HCs, prone to interaction with APOL3 HC2 [8]. As the APOL3 HC2 is involved in APOL3 binding to NCS‐1, APOL1/APOL3 interaction interferes with APOL3/NCS‐1 interaction, explaining the inactivation of PI4KB that occurs under these conditions (Fig. 4).

Whether C‐terminal variants of APOL1 interact with APOLs distinct from APOL3 is currently unknown, but the lower apoptotic potential of APOL1Δ cells as compared to APOL3KO cells [8] suggests that APOL1 variants could inactivate other death‐promoting proteins than APOL3.

### Relevance of the model to kidney disease

Several observations support the conclusion that the model presented in Fig. 4 [8] likely accounts for the APOL1 involvement in kidney disease.

- In keeping with this model, kidney disease induced by APOL1 variants in transgenic mice involved modifications of podocyte vesicular traffic and autophagy [4]. This phenotype, also observed in podocytes isolated from G1, G2 or G1/G2 patients, contrasts with the cytotoxicity reported for APOL1 variants in various experimental studies involving ectopic APOL1 (over)expression in heterologous assay systems [13, 14, 62]. APOL1 cytotoxicity could result from experimental artefacts such as protein mislocalization [15].
- Significantly reduced levels of PI(4)P were measured not only in different podocyte cell lines of the G1, G2 or G1/G2 genotypes, but also in glomeruli of kidney biopsies from human G1 or G2 patients [8].
- Kidney disease is not only genetically linked to APOL1 C‐terminal variants, but also to natural APOL3 KO [63]. Since natural APOL1 KO does not induce kidney disease [60], the most conservative explanation of these different observations is a negative effect of the APOL1 variants on APOL3.
- The proposal that increased APOL1 hydrophobicity due to exposure of HCs accounts for kidney disease easily explains the puzzling observation that recruitment of the APOL1 C‐terminal variants to lipid droplets attenuates cell toxicity [64].
- The reduction of Golgi PI(4)P levels and consecutive actomyosin reorganization can account for the recent report of APOL1 variant‐linked changes in cationic fluxes at the plasma membrane [62], because reducing either Golgi PI(4)P levels or NM2A activity can affect cation transport at the cell surface [65]. However, these changes more likely result from experimental artefacts due to improper APOL1 variant localization [15].
- The proposal that APOL1‐induced kidney disease is due to actomyosin reorganization is in agreement with distinct instances of kidney disease that are linked to mutations in other actomyosin components [66, 67]. Given the particularly important cytoskeleton constraints required for the biology of podocytes, such as the architecture of pedicels, the high susceptibility of these cells to actomyosin changes can readily be explained.
- The proposal that NM2A is both associated with and controlled by APOL1 harks back to the initial proposal that the motor domain of NM2A (MYH9) is linked to kidney disease prevalent in individuals of African origin [68, 69]. In this respect, the evolutionary conserved linkage between the contiguous APOL locus and MYH9 gene is worth mentioning.
- The increased organelle fission observed in APOL1Δ and APOL3KO podocytes [8] strikingly parallels the inhibition of mitochondrial fission observed in trypanosomes with ingested APOL1 [10], proving that podocyte actomyosin activity changes such as increased fission can indeed result from APOL inactivation. Accordingly, a recent report concluded that the G1 and G2 variants, but not wild‐type APOL1, trigger mitochondrial fission [70]. Thus, the fission inhibitory activity of APOLs is lost with the G1 and G2 variants like it is with APOL1Δ. Since in podocytes, the fission inhibitory activity was lost following APOL3 KO but not APOL1 KO, in these cells contrary to trypanosomes, the APOL1 potential to inhibit mitochondrial fission is inactive, and increased fission is strictly related to reduction of Golgi PI(4)P levels. That reduction of PI(4)P at the Golgi could trigger mitochondrial fission may occur through delocalization of Golgi PI(4)P‐binding proteins, such as GOLPH3, to ER/mitochondrion contact sites (MERCs; see below).

Another issue pertains to the linkage between APOL1‐induced kidney disease and viral infection [7]. In a recent case report, viral infection was present in four out of five cases of kidney disease resulting from transplantation of kidneys with APOL1 risk variants, highlighting the importance of virus presence as ‘second hit’ leading to glomerular injury [71]. This second hit is likely not a viral component *per se*, but rather the inflammation caused by viral infection. Indeed, kidney disease linked to APOL1 variants can also be promoted by the autoimmune disease systemic lupus erythematosus, which involves type I interferon‐mediated inflammation like viral infection [72]. In cultured podocytes, activation of inflammation by the viral mimetic poly(I:C) strongly increased expression of APOL1, APOL1 variants and APOL3 [8]. In wild‐type podocytes, poly(I:C) also increased the Golgi APOL1/APOL3 ratio and induced a reduction of the APOL3 amount at the Golgi [8]. Although from the model this last effect could be expected to reduce PI4KB activity due to a lower level of PI4KB‐stimulating APOL3 at the Golgi, no major difference in cellular PI(4)P level was observed [8]. I propose that interferon signalling induces PI4KB delocalization from the Golgi to the ER, like occurs following nutrient depletion for both PI4KB and PIK1/FRQ1 together with the reverse shuttling of the PI(4)P phosphatase suppressor of actin 1 (SAC1) from the ER to the Golgi [57, 73]. Indeed, interferon signalling and energy deprivation share the characteristic of triggering autophagy [74, 75], and several interferon‐inducible Golgi GTPases drive the movement of Golgi membranes, with proteins such as APOL2, to MERCs [76, 77, 78]. Such inflammation‐induced delocalization of PI4KB‐APOL3 complexes from the Golgi to MERCs could participate in the initiation of autophagy [56] and eventually phagophore elongation from mitochondrial membranes [59]. The interference of the APOL1 variants with inflammation‐induced autophagy could accentuate kidney pathology, because autophagy is vital in podocytes where the rate of cellular renewal is low.

Another contribution of viral infection to kidney disease could result from the strong increase of APOL1 transcripts that is induced by inflammation [5, 6, 8, 10]. Indeed, due to their altered secondary structure with respect to that of wild‐type APOL1 transcripts, transcripts for C‐terminal APOL1 variants can trigger the toxic activity of double‐stranded RNA‐dependent protein kinase R [79], and this toxicity would be enhanced with increased RNA amounts. Following this hypothesis, kidney disease linked to APOL3 KO would not be expected to exhibit association with viral infection.

### Nonrenal pathologies linked to the APOL1 variants

In addition to the risk of kidney disease, the C‐terminal APOL1 variants were significantly associated with other pathologies. A linkage with increased cardiovascular mortality was observed, but this could occur through the effects of the APOL1 variants on kidney disease itself [80]. Either fetal or maternal expression of the APOL1 variants was significantly linked to maternal preeclampsia, likely through trophoblast dysfunction [81, 82]. In this regard, when expressed as transgenes in mice, both wild‐type APOL1 and the APOL1 variants triggered preeclampsia, but with less severity in case of the wild‐type [83]. This observation evokes the differential level of interaction occurring between the different APOL1 versions and APOL3 [8], suggesting that APOL3 inactivation could be responsible for preeclampsia. The involvement of APOL3 in the control of cellular adherence and motility [8] might possibly account for altered trophoblast invasion linked to preeclampsia. Finally, significant association was also noted between expression of the APOL1 variants and sepsis [84, 85]. The involvement of APOL1 in sepsis could be related to the observed correlation between the expression levels of APOL1, but not APOL3, and apoptosis of neutrophils in critically ill patients [86]. Since delayed neutrophil apoptosis participates in sepsis, the reduction of apoptotic potential of C‐terminal APOL1 variants [8] provides an explanation for the increase in sepsis linked to G1 or G2 expression.

## APOLs and neurotransmission disorders

The strong interaction of APOL3 with NCS‐1 not only associated APOL3 with the control of PI4KB, but also associated APOLs with neurotransmission since NCS‐1 is heavily involved in secretion and exocytosis, particularly in neurones [28]. This involvement results from NCS‐1 control activities not only on PI(4)P, but also on Ca^2+^ levels. NCS‐1 activates Ca^2+^ efflux from intracellular stores through either direct binding to IP3R channels [87] or through indirect effects of PI(4)P on levels of IP3, which stimulates the activity of IP3R channels. Accordingly, numerous studies provided genetic and experimental evidence for the involvement of NCS‐1 in several neurological disorders such as bipolar disorder, schizophrenia and autism [88, 89, 90, 91, 92, 93, 94] (review in Ref. [95]). Moreover, in addition to the relationship between the PI4KB activator NCS‐1 and schizophrenia, genetic and functional linkages were also observed between the PI4KB inhibitor CALN‐1 and schizophrenia [96, 97, 98].

In this context, it is therefore worth mentioning that significant genetic association was reported between APOL genes and schizophrenia [99, 100] (review in Ref. [101]) or bipolar disorder [102]. Along the same line, the increase in APOL2 RNA was among the most significant transcriptional results of drug abuse [103], APOL3 was a good predictor of stress‐linked depression and exhibited significant expression decrease both in the brain of mice subjected to stress and in blood biomarker studies of suicide [104], and APOL6 expression was the second most highly up‐regulated in connection with HIV‐associated neurocognitive disorders [105].

Given these considerations, whether neurotransmission is affected by the G1 or G2 APOL1 variants would clearly be interesting to analyse.

## APOLs and cancer

The phenotype of APOL1Δ and APOL3KO podocytes exhibits striking similarities to that resulting from cancer metastasis, with strong reduction of cellular adherence and increase in cell motility, together with important reduction of the capacity for apoptosis [8]. Accordingly, the APOL3‐controlled NCS‐1 is known to promote motility, metastatic spread and survival of cancer cells [106, 107], and the NCS‐1 antagonist CALN‐1 also exhibits relationship with metastatic cell migration [108]. Since in human breast cancer, Golgi PI(4)P is known to regulate cellular adhesion and invasive cell migration [109], the observed reduction of PI(4)P in APOL1Δ and APOL3KO podocytes could be responsible for this metastasis‐like phenotype. More specifically, such phenotype could result from the decrease in GOLPH3 association with *trans*‐Golgi membranes due to the reduction of PI(4)P levels. Indeed, GOLPH3 is the only known oncogene of the Golgi and is suspected to play a role in metastasis of multiple tumour types including 52% of breast cancers and 41–53% of glioblastoma [50, 110, 111].

In keeping with these observations, APOLs were suspected to be involved in several cancers, including cervical, ovarian, breast, thyroid, bladder, prostate and colorectal cancers [12, 112, 113, 114, 115, 116].

## APOLs and viral infection

The strong induction of APOL expression resulting from viral activation of the TLR3/TRIF pathway is probably meant to trigger apoptosis of virus‐infected cells [6, 8]. Activation of this pathway induces autophagy [6,^74^], and autophagy is involved in APOL‐mediated apoptosis [6, 117]. The involvement of APOLs in autophagy notably occurs through their control of PI4KB, which is a key component in the initiation of autophagy [56] (Fig. 3). PI4KB is also crucially involved in the PI(4)P‐dependent remodelling of cellular membranes induced by different viruses in order to build membranous structures such as replication organelles or inclusion bodies, where new virions are assembled [118, 119, 120, 121, 122, 123, 124, 125, 126, 127, 128, 129, 130] (review in Ref. [131]). Such is particularly the case for various picornaviridae, which recruit PI4KB by hijacking different PI4KB‐binding proteins such as ACBD3 or c10orf76 [119, 122, 123, 125, 126, 128, 129, 130]. Other viruses, such as hepatitis C, coronavirus or parainfluenza type 3, also depend on PI4KB for their replication and secretion [118, 120, 121, 123, 127]. Finally, flaviviruses also trigger autophagy‐related intracellular membrane remodelling for the building of membranous replication platforms, which involves strong alteration of lipid composition that includes the increase of PI [131, 132, 133]. Therefore, PI(4)P and its synthesizing enzyme PI4KB are considered as pan‐viral requirements for virus replication [134]. However, despite this evidence it is not known whether the PI4KB controllers NCS‐1 and CALN‐1 participate in viral replication, and whether the effects of APOLs on viral infection involve the control of PI4KB.

In high‐throughput screenings of type I interferon‐stimulated genes, APOLs have emerged as antiviral proteins with a large spectrum of activities, acting against the Sindbis virus, encephalitis virus, parainfluenza virus type 3, hepatitis C virus, picornaviruses, coxsackie B virus, poliovirus and respiratory syncytial virus [135]. However, in the same study some APOLs also exhibited proviral activity, promoting infection of the yellow fever virus, influenza A virus and respiratory syncytial virus [135]. In none of the listed cases were the mechanisms involved in APOL effects on viral infection deciphered. In macrophages cultivated *in vitro*, APOL1 restricted HIV‐1 replication [9]. Such effect was not observed in HIV‐1‐infected individuals expressing the APOL1 G1 and G2 variants [136]. In cultured mouse fibroblasts, mAPOL9, which is induced by type I interferon as occurs for human APOL1 and APOL3, was found to specifically restrict replication of the Theiler's murine encephalomyelitis virus (TMEV), and this involved mAPOL9 interaction with prohibitins (PHB) [137]. Since PHB 2 targets mitochondria for autophagic degradation [138], mAPOL9 could be involved in autophagy. PHB were also detected among the proteins immunoprecipitated with anti‐APOL1 or anti‐APOL3 antibodies in human podocyte extracts ([8] and Uzureau S & Pays E, unpublished). Therefore, in macrophages PHB could be involved in the APOL1 restriction of HIV‐1 replication, which results from increased endolysosomal protein degradation resembling autophagy [9]. Conversely, PHB 2 was found to favour infection by Enterovirus A71 through the formation of autophagy vesicles acting as replication scaffolds [139]. Thus, the autophagy‐linked activities of PHB 2 exerted opposed effects on infection by two different picornaviruses, TMEV and Enterovirus A71 [137, 139]. PHB also promoted infection by other viruses such as the DENV‐2 flavivirus and Chikungunya togavirus [140, 141]. Interestingly, like PHB mAPOL9 exerted opposite autophagy‐linked effects on viral infection. Indeed, besides its inhibitory effect on TMEV, mAPOL9 conversely favoured replication of the Japanese encephalitis flavivirus [142], and mAPOL9 was associated with autophagosome components [143]. Thus, PHB and mAPOL9 are interacting proteins that either promote or inhibit viral replication depending on the nature of the virus, and their activities are both associated with autophagy.

**Fig. 5:**
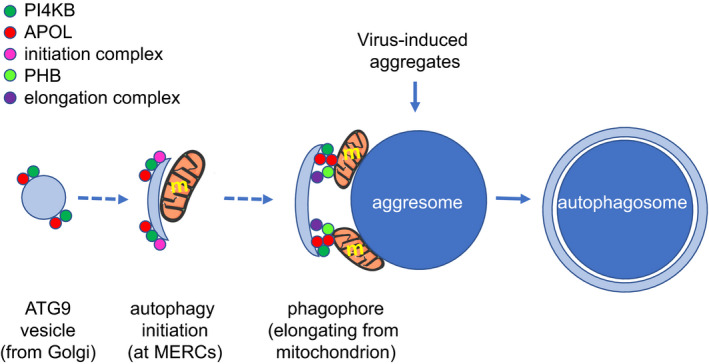
Hypothetical involvement of APOLs in the formation of autophagosomes induced by type I interferon. Aggresomes are virus‐induced aggregates that become enclosed in autophagic structures to limit infection and favour cell recovery [145], but such structures may be used by some viruses for their replication [131, 147]. APOLs could control the initiation of isolation membranes by Golgi‐derived vesicles, owing to their effect on PI(4)P synthesis by PI4KB [8], which is key to autophagy initiation [56, 166]. In addition, APOLs could contribute to the process by which mitochondria donate their membranes to phagophores [146, 170], owing to their association with both the autophagy receptors PHB and autophagy factors LC3/GABARAP, together with their common binding to cardiolipin [8, 117, 137, 143, 162]. Since Golgi‐derived PI4KB‐containing vesicles play a part in the fission of mitochondrial membranes at MERCs [59], where autophagy and mitophagy are initiated [174], the succession between the initiation and elongation processes depicted here can be envisaged (dotted arrow). m, mitochondrion.

While PHB and APOLs seem to be part of an autophagy system linked to infection, PI4KB is likely to be associated with this system given involvement of this enzyme in both autophagy and viral replication [56, 118, 119, 120, 121, 122, 123, 124, 125, 126, 127, 128, 129, 130, 131]. Accordingly, PI4KB was found to be involved in autophagy linked to infection by group A *Streptococcus* [144]. Therefore, I propose that APOLs, PI4KB and PHB participate in the formation of autophagosomes induced by infection, particularly by viruses. More specifically, these proteins would generate and elongate phagophores enclosing virus‐induced aggresomes for their degradation in autophagosomes [145] (Fig. 5). While APOLs control the activity of PI4KB that contributes to initiate the formation of isolation membranes [56], the participation of APOLs in mitophagy‐linked phagophore elongation [138, 146] could result from their control of the PI4KB involvement in mitochondrial membrane fission at MERCs [59], as well as from their interaction with PHB and autophagy proteins [137, 143]. Viral escape from such mechanism would involve PI4KB hijacking to build alternative membranous replicative platforms [131], or the use of aggresomes and autophagosomes to generate replication sites [147].

In conclusion, it would seem as if APOLs were evolutionary generated as instruments to limit viral infection through their involvement in PI4KB‐dependent autophagy and autophagy‐related apoptosis, following their strong induction by the virus‐activated TLR3/TRIF pathway. However, several viruses managed to hijack PI4KB activity for their own replication. Therefore, APOLs could appear as either anti‐ or proviral factors, depending on the virus type.

## Concluding remarks

### The various APOL activities: links and effects on mitochondrial fission, apoptosis and autophagy

Despite their overall sequence conservation, the six members of the human APOL family appear to play different functions. Among these proteins APOL1 and APOL3 standout, both are trypanolytic and the expression of both these APOLs is increased massively in response to poly(I:C) [6, 11]. APOL2 appears to be devoid of apoptotic potential [148], and indeed, this protein did not seem to contribute to induction of cell death by poly(I:C) [8]. On the contrary, in human bronchial epithelium APOL2 exhibited antiapoptotic ability [149]. APOL6 triggered apoptosis upon ectopic overexpression [150], but given the lack of APOL6 trypanolytic activity [11], this death‐promoting activity could result from nonspecific toxicity as was also observed under the same conditions for APOL1 [15]. The function of APOL4 and APOL5 is unknown, apart from the lack of apoptotic activity for APOL4 [117] and the genetic association of APOL4 with schizophrenia [99, 100, 101].

APOL1 and APOL3 appear to be involved in the control of Golgi PI(4)P synthesis in kidney podocytes [8], but the relevance of this observation regarding the mechanisms controlling PI4KB in other cell types, as well as regarding the precise processes underlying PI4KB activation in general, is unknown. Through its high‐affinity and Ca^2+^‐dependent interaction with factors that control PI4KB activity (NCS‐1 and CALN‐1), APOL3 has the capacity to affect PI(4)P synthesis. However, provided APOL1 is absent, PI4KB remains active in APOL3 KO podocytes, suggesting that NCS‐1‐mediated PI4KB activation can occur independently from APOL3 [8]. Moreover, NCS‐1 can stimulate *in vivo* activity of the yeast PI4KB homologue (PIK1) in the apparent absence of any yeast homologue of APOL3 [34]. Therefore, APOL3 is clearly not obligatory for *in vivo* PI4KB activity.

Whether the ion transport activity of APOL1 and APOL3 is related to PI4KB control is unclear. In this respect, it should be noted that the two APOL3‐interacting proteins, NCS‐1 and CALN‐1, not only control PI4KB [40], but also influence the level of Ca^2+^ in intracellular stores [28, 43]. Furthermore, APOL1 and APOL3 also influence Ca^2+^ storage [8]. Such activity, operating at the cytosolic periphery of *trans*‐Golgi and ER membranes, is obviously relevant for PI4KB control, which is known to be Ca^2+^‐dependent. Therefore, I propose that APOL3 and NCS‐1 are responsible for conferring Ca^2+^‐dependent stimulation of PI4KB activity at the Golgi. However, PI4KB could be active independently of this context, as observed in APOL3+1KO podocytes. Such NCS‐1‐ and Ca^2+^‐independent activity could possibly be at work following PI4KB hijacking by some viruses.

Whether the ion transport activity of APOL1 and APOL3 is related to their apoptotic potential is also unclear. It may be relevant that a similar ion channel activity is also present in apoptotic BCL2 proteins [12]. Transmembrane ion flux could be linked to an additional and important characteristic shared between APOL1, APOL3 and the BCL2 family members, namely their control of mitochondrial fission [8, 10, 69, 151, 152]. Both APOL1 and APOL3 can inhibit mitochondrial fission, although in podocytes this function is only exerted by APOL3 [8, 10, 70]. The relationship between mitochondrial fission and apoptosis is well established [153]. In both processes, cardiolipin appears to play a key role. Cardiolipin mediates the recruitment at mitochondrial fission sites, of both the DRP1 dynamin essential for fission and the BCL2 family members essential for apoptosis [154, 155, 156, 157, 158, 159, 160]. Therefore, the ability of APOL1 and APOL3 to strongly bind cardiolipin [8, 112, 117] could explain their effect on mitochondrial fission and apoptosis. In addition, Golgi PI(4)P and phosphatidic acid are also involved in mitochondrial and Golgi fission, respectively [23, 59]. I propose that the effects of cardiolipin, phosphatidic acid and/or PI(4)P on membrane remodelling, deformation and permeabilization [21, 22, 23, 24, 44, 45, 161] are involved in the insertion of APOL1 and APOL3 into mitochondrial and Golgi membranes (Fig. 2). Thus, the potential of APOL1 and APOL3 to inhibit mitochondrial fission and trigger apoptosis could tentatively be related to their abilities to bind cardiolipin, phosphatidic acid and PI(4)P.

The association of APOLs with cardiolipin, mitochondrial fission and apoptosis also connects APOLs with PHB. Indeed, mAPOL9 interacts with PHB [137] and PHB are present in APOL1 and APOL3 immunoprecipitates ([8] and Uzureau S & Pays E, unpublished). PHB are mainly found in the mitochondrial inner membrane, where they interact with cardiolipin and promote mitochondrial fusion [162], while also behaving as receptors for mitophagy [138] and inducing autophagy upon interaction with some viruses [139] (review in Ref. [163]), all features evoking APOL1 and APOL3 characteristics. Moreover, minor amounts of PHB are found at the Golgi membrane, where they appear to be involved in membrane trafficking between the Golgi and the ER [164]. In this context, it is worth mentioning that in yeast, PHB may interact with the PI(4)P phosphatase SAC1 [164]. This is relevant to autophagy control, because following autophagy induction by nutrient starvation, SAC1 moves from the ER to the Golgi in inverse movement of PI4KB from the Golgi to the ER [73]. Also worth mentioning, PHB confer strong protection against neuronal injury in models of cerebral ischaemia [162], evoking promotion of autophagy, which would also contribute to explain the protection afforded by the APOL‐related APOLd1 against ischaemic stroke [165], assuming that PHB act together with APOLd1. Therefore, PHB seem to affect autophagy in association with the APOL‐PI4KB activity complex.

I propose that APOLs, PI4KB and PHB participate in the formation of autophagosomes induced by type I interferon‐mediated inflammation [145] (Fig. 5). This model is inspired by the effects of nutrient starvation in both yeast and mammalian cells. In both cases, nutrient starvation triggers a reduction of Golgi PI(4)P levels due to PI4KB release from the Golgi combined with import of the PI(4)P phosphatase SAC1 at the Golgi [57, 73], and this is linked to induction of autophagy at MERCs, which requires the activity of PI4KB in Golgi‐derived vesicles [56, 166]. The transfer of PI4KB‐containing vesicles from the Golgi to MERCs involves NM2A [56, 167, 168], which is tightly associated with APOLs [8]. In podocytes, the reduction of Golgi PI(4)P levels due to APOL3 inactivation was linked to increased mitochondrial fission [8], which is involved in autophagy triggered by energy deprivation [1699]. Like occurs following nutrient starvation, cell activation by poly(I:C) also involves the induction of autophagy at MERCs [74, 75], which could be coupled to a reduction of PI4KB levels at the Golgi since poly(I:C) triggers a reduction of Golgi APOL3 levels [8]. In addition to controlling PI4KB activity at *trans*‐Golgi ‐derived vesicles targeted to MERCs, APOLs could also play a role in phagophore elongation coupled with mitophagy [146, 157, 170]. Indeed, PI4KB and PI(4)P of *trans*‐Golgi‐derived vesicles are required for the fission of mitochondrial membranes [59], maybe in association with the membrane‐deforming and PI(4)P‐binding protein GOLPH3, which shuttles between the Golgi and mitochondrion for cardiolipin delivery [171, 172]. Moreover, APOLs are not only localized at the Golgi and the ER, but they also appear to be recruited to specific sites of the mitochondrial membrane, such as MERCs: (a) in both trypanosomes and podocytes, a fraction of APOL1 was detected on restricted sites of the mitochondrion [8, 10, 173], (b) APOL2 moves to MERCs during viral infection [78], and (c) APOLs interact with the mitophagy components cardiolipin, PHB and some autophagy proteins [8, 112, 137, 143]. Thus, maybe under certain conditions like inflammation, APOL1, APOL2 and APOL3, and possibly also APOLd1, concentrate at MERCs where fission, autophagy and apoptosis can be triggered [174]. While APOL1 and APOL3 can inhibit fission, promote autophagy and promote apoptosis, APOL2 and APOLd1 would trigger nonapoptotic autophagy that protects epithelial and endothelial cells from inflammation‐ or hypoxia‐linked cytotoxicity [149, 165]. In addition, APOL3 could be involved in the recruitment of the proteasome for Parkin‐induced degradation of the outer mitochondrial membrane before phagophore interaction with PHB, since in yeast two‐hybrid screens [8], APOL3 was found to interact both with the proteasomal non‐ATPase regulatory subunit 2 (PMSD2), which directly binds to the core autophagy protein ATG16 [175], and with the ubiquitin E3 ligase tripartite motif‐containing protein 8 (TRIM8), which regulates autophagy [176, 177].

### APOL1 specificity

Given the high sequence and structure similarity between APOL1 and APOL3, it is remarkable that only APOL3 can interact with NCS‐1 and CALN‐1. Conversely, in addition to its selective capacity to be secreted, APOL1 exhibits specific potential for *cis*‐interactions, which is not found in APOL3. Apart from these differences, in podocytes APOL1 exerted apoptotic and Ca^2+^ storage activities seemingly redundant with those of APOL3, APOL1 did not share the APOL3 inhibitory activity on mitochondrial fission, and more generally, APOL1KO cells exhibited a wild‐type‐like phenotype. Accordingly, human individuals naturally lacking APOL1 are healthy [61]. Thus, APOL1 is clearly dispensable. Given the evolutionary recent appearance of APOL1 in primates, it is tempting to propose that APOL1 emerged by gene duplication only for the purpose of resisting African trypanosomes in the bloodstream. This hypothesis would readily explain all APOL1 original features with respect to APOL3. On the one hand, the necessity of meeting trypanosomes in the blood obviously accounts for the first insertion of a SP in the history of the APOL family. So far, there is no clear evidence for another function of APOL1 in the serum. In particular, the correlation between APOL1, triglycerol levels and hyperglycaemia only reflects APOL1 association with very low‐density lipoprotein particles, without known causality relationship [178]. On the other hand, the specific dual pH dependence of the APOL1 channel (first requirement of acidic pH for transmembrane insertion and second requirement of neutral pH for efficient cation transport) is difficult to explain unless it results from constraints for APOL1 transport and lytic activity in trypanosomes (first endocytic pathway, then mitochondrial membrane). Finally, regarding *cis*‐interactions, they are not required for pore‐forming activity, but given the cellular dysfunction resulting from the reduction of the *cis*‐interactions in C‐terminal APOL1 variants [8], it is reasonable to propose that they were meant to prevent pathological interference of intracellular APOL1 with the functions exerted by APOL3. Due to the involvement of the LZ2 helix in both APOL1 *cis*‐interactions and SRA binding, LZ2 mutations designed to avoid APOL1 neutralization by SRA (G1 and G2) also implied some reduction of *cis*‐interactions, progressively triggering *trans*‐interactions with APOL3 and APOL3 inactivation, hence kidney disease. Obviously, human evolution in Africa favoured resistance to sleeping sickness over progressive induction of kidney disease, and kidney disease could be considered as a price to pay for resistance to *T. rhodesiense*. Given its pathogenic potential, the evolutionary conservation of intracellular APOL1 despite its dispensable nature is intriguing. Insufficient counterselection could be due to the late appearance of the risk variants in human evolution or late appearance of the disease during life.

## Conflict of interest

The author declares no conflict of interest.
